# Stress-induced decreases in local cerebral glucose utilization in specific regions of the mouse brain

**DOI:** 10.1186/1756-0500-4-96

**Published:** 2011-03-31

**Authors:** Geoff I Warnock, Thomas Steckler

**Affiliations:** 1 University of Liege, Cyclotron Research Center, Allée du 6 Août, 8, 4000 Liege, Belgium; 2 Dept. Psychiatry, RED Europe, Johnson & Johnson PRD, Turnhoutseweg 30, B-2340 Beerse, Belgium

## Abstract

**Background:**

Restraint stress in rodents has been reported to activate the hypothalamic-pituitary-adrenocortical (HPA) axis and to increase c-fos expression in regions that express components of the corticotropin-releasing factor (CRF) system. We have previously reported that acute central administration of CRF increased a measure of relative local cerebral glucose utilization (LCGU), a measure of neuronal activity in specific brain regions, and activated the HPA axis in mice. It was hypothesized that the involvement of the CRF system in the stress response would lead to similar changes in relative LCGU after restraint stress. In the present studies the effect of restraint stress on relative LCGU and on the HPA axis in C57BL/6N mice were examined.

**Findings:**

Restraint stress activated the HPA axis in a restraint-duration dependent manner, but in contrast to the reported effects of CRF, significantly decreased relative LCGU in frontal cortical, thalamic, hippocampal and temporal dissected regions. These findings support evidence that stressors enforcing limited physical activity reduce relative LCGU, in contrast to high activity stressors such as swim stress.

**Conclusions:**

In conclusion, the present studies do not support the hypothesis that stress-induced changes in relative LCGU are largely mediated by the CRF system. Further studies will help to delineate the role of the CRF system in the early phases of the relative LCGU response to stress and investigate the role of other neurotransmitter systems in this response.

## Background

Restraint stress has been reported to activate the hypothalamic-pituitary-adrenocortical (HPA) axis [1-6], and has also been reported to increase c-fos expression in regions that express components of the corticotropin-releasing factor (CRF) system [7-10]. We have previously reported that acute central administration of CRF increased a measure of relative local cerebral glucose utilization (LCGU) [11], a measure of neuronal activity in specific brain regions [12]. Therefore, it was hypothesized that the involvement of the CRF system in the stress response would lead to similar changes in relative LCGU after restraint stress. The aim of the present study was to examine the effects of restraint stress on relative LCGU.

To study the effects of restraint stress on relative LCGU and the HPA axis, relative LCGU and plasma corticosterone were measured after various durations of restraint stress. Furthermore, to verify that the tracer dose of 2DG used to measure relative LCGU was well below the threshold for activation of the HPA axis, the effect of 2-Deoxy-D-glucose on plasma corticosterone was measured independently.

## Methods

Male C57BL/6N mice (Janvier, France; mean body weight at testing 26.9 ± 1 g) were individually housed in individually-ventilated cages with food and water available ad libitum (12 h/12 h light-dark cycle (lights on 06:00 hours), temperature 22 ± 0.5°C, humidity of 50 ± 3%). All testing (randomized, between 07:00 and 12:00 hours) was conducted according to the European Communities Council Directive Nov. 1986 (86/609/EEC) and was approved by the animal care and use committee of Johnson & Johnson Pharmaceutical Research & Development.

For the measurement of relative LCGU, 2-deoxy-D-[1-^3^H]glucose (2DG; GE Healthcare, UK) (300 μCi/kg) was injected intraperitoneally immediately before restraint stress. Although this modified version of the LCGU technique does not account for inter-subject variability as comprehensively as the original technique with repeated blood sampling, it minimizes both stress and effects on the animals' physiology induced by blood sampling. For the restraint stress, mice were removed from their home cage, injected with 2DG then placed in well ventilated stainless steel restraining tubes (9 cm length, 3 cm diameter) for between 2 and 45 minutes. Mice receiving restraint stress for less than the full 45 minute duration of the LCGU protocol were returned to their home cage for the remainder of the protocol duration. 45 minutes after 2DG injection, blood and brain were collected for analysis.

Trunk blood was collected in BD Microtainer K2E tubes (BD Vacutainer Systems, UK), 10 μl of whole blood was used to measure blood glucose using a Lifescan glucose meter (Lifescan Benelux, Belgium), and the remainder was centrifuged at 1100 g for 10 minutes to separate plasma from red blood cells. Plasma samples were collected for measurement of plasma ^3^H (residual 2DG) and corticosterone and stored at -80°C.

After blood collection the brains were dissected into frontal cortical area (anterior to corpus callosum), hypothalamus, thalamus, cerebellum, hindbrain (a block defined from the colliculi to the posterior level of the cerebellum), hippocampus and temporal region (including the amygdala). Tissue samples were weighed, and stored at -80°C until homogenization. Brain samples were dissolved using Solvable tissue solubilizer (Perkin Elmer, Belgium). ^3^H in plasma and brain samples was measured in duplicate in a TopCount NXT (PerkinElmer, Belgium) microplate scintillation counter after addition of Microscint-40 scintillant (PerkinElmer, Belgium). A relative measure of LCGU was calculated as nCi ^3^H present per mg of brain tissue.

To verify that the tracer dose of 2DG used to measure relative LCGU had no effect on the HPA axis, the effect of 2-Deoxy-D-glucose on plasma corticosterone was measured. 2-Deoxy-D-glucose (non-radiolabelled; Sigma Aldrich, Germany) was injected intraperitoneally at doses from 5-400 mg/kg in saline vehicle. After 45 minutes plasma was collected as described above for the measurement of plasma corticosterone. Plasma corticosterone was measured using ImmuchemTM Double Antibody corticosterone ^125^I RIA kits (MP Biomedicals, USA).

The data were statistically analyzed using the Kruskal-Wallis (KW) test with post-hoc comparisons performed by the Mann-Whitney (MW) test (SPSS v13.0, SPSS Belux, Belgium) at a significance level of p = 0.05.

## Results

45 minutes of restraint significantly reduced relative LCGU (nCi/mg) in the frontal cortical (p = 0.007), thalamic (p = 0.001), hippocampal (p = 0.006) and temporal (p = 0.034) dissected regions (Table 1). The largest decrease was seen in the thalamic region in which relative LCGU was reduced from 0.22 ± 0.02 nCi/mg to 0.15 ± 0.01 nCi/mg. Relative LCGU was not significantly reduced by shorter durations of restraint. No changes in final plasma ^3^H levels (residual plasma 2DG) or blood glucose were found after any duration of restraint stress studied (Table 2). No relationship was found between blood glucose and relative LCGU in any of the altered regions. Plasma corticosterone levels were increased in a restraint-duration dependent manner (Table 2) from 2 min (p = 0.016) to 5 min (p = 0.019), 10 min (p = 0.006), 20 min (p = 0.001) and 45 min (p < 0.001). Control plasma corticosterone levels were 65 ± 15 ng/ml and the maximum level after 45 minutes of restraint stress was 323 ± 11 ng/ml.

**Table 1:** The effect of different durations of restraint stress on relative LCGU in mice (n = 8-10) (*p < 0.05)

Restraint Stress	Relative LCGU (nCi/mg)					
						
Region	Control	2 min	5 min	10 min	20 min	45 min
**Frontal Cortical**	0.27 ± 0.02	0.24 ± 0.03	0.22 ± 0.01	0.30 ± 0.02	0.24 ± 0.01	0.21 ± 0.02 *
**Hypothalamus**	0.20 ± 0.01	0.19 ± 0.02	0.18 ± 0.01	0.22 ± 0.01	0.20 ± 0.02	0.18 ± 0.01
**Thalamus**	0.22 ± 0.02	0.19 ± 0.02	0.18 ± 0.02	0.24 ± 0.02	0.20 ± 0.02	0.15 ± 0.01 *
**Cerebellum**	0.13 ± 0.01	0.11 ± 0.02	0.12 ± 0.02	0.13 ± 0.02	0.10 ± 0.01	0.09 ± 0.01
**Hindbrain**	0.10 ± 0.01	0.08 ± 0.01	0.08 ± 0.02	0.09 ± 0.01	0.07 ± 0.01	0.06 ± 0.01
**Hippocampus**	0.15 ± 0.01	0.13 ± 0.01	0.13 ± 0.01	0.16 ± 0.00	0.14 ± 0.01	0.12 ± 0.01 *
**Temporal**	0.18 ± 0.01	0.15 ± 0.01	0.15 ± 0.01	0.25 ± 0.06	0.16 ± 0.01	0.15 ± 0.01 *

**Table 2:** The effect of different durations of restraint stress on residual plasma 2DG, blood glucose and plasma corticosterone in mice (n = 8-10) (*p < 0.05)

Treatment	Residual plasma 2DG (nCi/ml)	Blood glucose (mg/dl)	Plasma corticosterone (ng/ml)
Control	44 ± 3	135 ± 9	65 ± 15
2 min	57 ± 12	163 ± 9	127 ± 23 *
5 min	57 ± 10	154 ± 12	112 ± 17 *
10 min	49 ± 3	136 ± 11	146 ± 23 *
20 min	50 ± 3	154 ± 8	188 ± 18 *
45 min	49 ± 6	144 ± 22	323 ± 11 *

**Figure 1:**
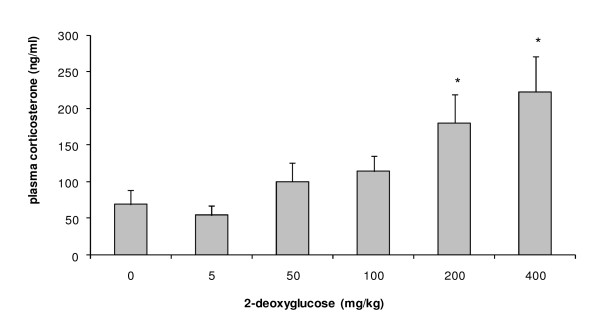
**The effect of intraperitoneally administered 2DG on the HPA axis**. (n = 6/group). *p < 0.05.

Administration of unlabelled 2DG resulted in a dose-dependent increase in plasma corticosterone after 45 minutes (Figure 1), only reaching statistical significance at the 200 and 400 mg/kg doses (p = 0.026 and 0.015) (equivalent to approximately 20 Ci/kg and 40 Ci/kg, respectively, when compared to 2DG used to measure relative LCGU at a typical specific activity of 8 Ci/mmol), more than one thousand-fold higher than the dose used to measure LCGU (approx 3 μg/kg at an injected dose of 300 μCi/kg).

## Discussion

The present studies aimed to examine the effects of acute restraint stress on relative LCGU, allowing a comparison to the effects of directly manipulating the CRF system on relative LCGU and the HPA axis previously reported [11].

In contrast to the increases in relative LCGU demonstrated after the administration of CRF [11], relative LCGU was reduced in selected brain regions after 45 minutes of restraint stress by up to 32% (in the thalamic region; Table 1). The opposing effects of restraint stress on relative LCGU compared to CRF administration suggest that restraint stress-induced changes in relative LCGU are unlikely to be mediated by a similar mechanism involving CRF. However, it is also possible that the relative LCGU decreases after restraint stress represent a prolonged response involving multiple neurotransmitter systems. This prolonged response may override initial effects of CRF release on relative LCGU. Reductions in LCGU have also been reported after stressors including an inactivity component such restraint in cold water and four-limb immobilization [13,14]. In the present study, the influence of the additional stress components, namely cold water and exposure during immobilization, in these studies was avoided, as conflicting effects on LCGU have been reported depending on the stressor studied. Indeed, increases in LCGU after swim stress have been reported in prefrontal cortical areas, motor cortex and lateral septum, and these effects matched with increased fos-like-immunoreactivity in these areas [15]. Reduced LCGU in the hippocampus, inferior colliculus, orbital cortex, and insula after swim stress has been reported, but LCGU was simultaneously increased in striatum and cerebellum [16,17]. Differences in the physical context of a stressor could explain contradictory findings for LCGU alterations. For example, restraint/immobility could be considered to reflect stressors with (imposed) limited physical activity, while clearly forced swim stress requires a large activity component. Interestingly, the forced swim stress-induced increase in LCGU in the lateral septum was blocked by treatment with imipramine [15], which in this acute context implicates the serotonergic and noradrenergic systems in these LCGU changes. However, swim-stress-induced changes in LCGU are also sensitive to temperature [18], meaning that care must be taken in comparing studies with different methodology.

Activation of the HPA axis after restraint stress (Table 2) agrees with reports from the literature [1-6], and is similar to that seen after central administration of CRF or the related endogenous peptides Urocortin 1, 2 and 3 [11]. These changes cannot be attributed to a direct effect of 2DG on the HPA axis, as plasma corticosterone was only increased at doses >100 mg/kg, while the tracer amount of 2DG used to measure relative LCGU equates to approximately 3 μg/kg. This agrees with previously reported data [19-21], in which 2DG was capable of activating the HPA axis at doses >100 mg/kg. The present study could have been improved by the inclusion of a dose of 2DG more closely matching the tracer dose used for LCGU measurements. Through competition with intracellular glucose, 2DG inhibits phosphohexose isomerase [22,23] and thereby blocks glycolysis at the initiation stage. Therefore, at sufficient doses, 2DG is expected to cause depletion of ATP as well as of glucose derivatives required for protein glycosylation [24], constituting a pharmacological stressor through energy depletion. The use of a tracer dose of 2DG which is only sufficient for the measurement of relative LCGU ensures that this effect is avoided in studies of central glucose metabolism.

Glucocorticoid feedback after activation of the HPA axis may play a role in LCGU changes during stress, particularly in regions associated with the HPA axis negative feedback loop, such as the hippocampus [25,26]. A classic catabolic action of glucocorticoids in numerous peripheral tissues is to inhibit glucose uptake into cells [27,28], and studies have shown that in hippocampal cell cultures glucocorticoids significantly inhibit glucose uptake and oxidation both by neurons and astrocytes [27,29], although exposure for at least four hours appears to be necessary. Increased LCGU following acute adrenalectomy in rats [30], further indicates a negative modulatory role of glucocorticoids on cerebral glucose metabolism, and an overall reduction in relative LCGU after direct administration of corticosterone has been reported [31], although the authors attributed the effect to peripheral changes in 2DG uptake. As similar evidence for a peripheral effect on 2DG uptake, as measured by changes in residual 2DG, was not found in the present study, the reductions in relative LCGU seen in the brain in the present study may be related to the increased corticosterone levels after restraint stress (Table 2). Despite this activation of the HPA axis, no significant effect on relative LCGU in the hypothalamic region was detected (Table 1). The heterogeneous nature of the overall hypothalamic region may explain the lack of a detected effect in this dissected region, although in a study with a similar methodology [31] an increase in hypothalamic relative LCGU was detected after footshock. Activation of the HPA axis would be expected to primarily involve the paraventricular nucleus [4,32], which comprises only a small fraction of the dissected region. This highlights a drawback of the present methodology. Further studies using higher resolution techniques, such as autoradiography would allow more detailed study of changes in the hypothalamus.

Restraint (and other stressors) has been reported to increase blood glucose levels [3,5,6,13,33]. As described in the original assumptions of the LCGU model [12], sufficient variations in blood glucose may influence the measurement of LCGU. For this reason, blood glucose was measured of as part of the relative LCGU protocol. No significant changes in blood glucose were found (Table 2), indicating that this variable should not have adversely affected relative LCGU measurements. However, a weakness of the present methodology is that an end point glucose measurement may miss transient changes during the 45 minute protocol. The absence of a change in blood glucose compared to studies in the literature may reflect strain differences as mice of a similar strain (C57BL/6J) were only used in one of the above studies [5]. Furthermore, in that study the mice were restrained for 1 hour, and shaken for 10 minutes during that period. This may constitute a much greater stressor than restraint alone, and the reduced duration of restraint stress used in the present study may contribute to the different findings.

In conclusion, the present studies do not support the hypothesis that restraint stress would induce similar changes in relative LCGU to those induced by CRF. However, the changes seen support evidence that stressors enforcing limited physical activity reduce LCGU globally.

## Competing interests

The authors declare that they have no competing interests.

## Authors' contributions

GW performed the studies, analysis, and wrote the manuscript. TS edited the manuscript and provided valuable scientific input. All authors read and approved the final manuscript.
